# Metformin regulates oxLDL-facilitated endothelial dysfunction by modulation of SIRT1 through repressing LOX-1-modulated oxidative signaling

**DOI:** 10.18632/oncotarget.7387

**Published:** 2016-02-14

**Authors:** Ching-Hsia Hung, Shih-Hung Chan, Pei-Ming Chu, Huei-Chen Lin, Kun-Ling Tsai

**Affiliations:** ^1^ Department of Physical Therapy, College of Medicine, National Cheng Kung University, Tainan, Taiwan; ^2^ Institute of Allied Health Sciences, College of Medicine, National Cheng Kung University, Tainan, Taiwan; ^3^ Department of Internal Medicine, National Cheng Kung University Hospital, College of Medicine, National Cheng Kung University, Tainan, Taiwan; ^4^ Department of Anatomy, School of Medicine, China Medical University, Taichung, Taiwan; ^5^ Department of Physical Therapy, Shu-Zen Junior College of Medicine and Management, Taiwan

**Keywords:** metformin, endothelial dysfunction, oxLDL, SIRT1, Gerotarget

## Abstract

It is suggested that oxLDL is decisive in the initiation and development of atherosclerotic injuries. The up-regulation of oxidative stress and the generation of ROS act as key modulators in developing pro-atherosclerotic and anti-atherosclerotic processes in the human endothelial wall. In this present study, we confirmed that metformin enhanced SIRT1 and AMPK expression in human umbilical vein endothelial cells (HUVECs). Metformin also inhibited oxLDL-increased LOX-1 expression and oxLDL-collapsed AKT/eNOS levels. However, silencing SIRT1 and AMPK diminished the protective function of metformin against oxidative injuries. These results provide a new insight regarding the possible molecular mechanisms of metformin.

## INTRODUCTION

Endothelial injuries motivated by oxidized low-density lipoprotein (oxLDL) acts a key role in atherosclerosis [1]. Oxidative stress is a continued level of oxidative damage in animal cells and is caused by an overabundance of reactive oxygen species (ROS) or a decline in antioxidant ability against them. Most importantly, the biological factors of ROS in the cardiovascular system are superoxide (O2^•^) and hydrogen peroxide (H2O2) [2]. They are generated in the vascular cells by a number of oxidases, such as the NADPH oxidases (Nox) and xanthine oxidase, lipoxygenases, cytochrome p450, uncoupling of the mitochondrial respiratory chain, and uncoupling of endothelial nitric oxide synthase (eNOS) [3]. Nitric oxide generated by nitric oxide synthase (eNOS) had been reported about its benefit for cardiovascular system by mitigating the platelet aggregation and formation of monocyte chemoattraction protein-1 (MCP-1). NO also attenuates the leukocyte-endothelium interaction and smooth muscle cell proliferation [4].

Essentially, SIRT1 (Sirtuin 1) acts an important role in regulating cellular physiological processes, such as metabolism, degeneration, growth and survival. In the human endothelial cells, SIRT1 regulates their anti-aging as well as protects against endothelial inflammation [5-7]. It is also shown to enhance antioxidant enzyme activity and inhibit free radical-mediated oxidative injuries *via* decreasing the NADPH oxidase activation [8, 9]. Cytoprotective function of SIRT1 had been reported, SIRT1 protects against lipid accumulation in hepatocytes [10]. Furthermore, a previous study reported the expression level and activity of SIRT1 that were reduced in the inflammatory endothelial cells [11]. Recently, SIRT1 is recognized as a novel target to prevent human endothelial pathology. For example, it protects against oxidative stress-induced ionomycin-induced ICAM-1 expression in endothelial cells[12]. Activating SIRT1 function through drugs are also reported to reduce oxidative injuries-induced endothelial cells death [13]. Moreover, the expression level of SIRT1 proves to decrease in the inflammatory human endothelial cells [11].

Clinically, metformin decreases both fasting and post-prandial blood glucose level, primarily through reducing hepatic glucose generation and possibly affects the peripheral glucose utilization [14, 15]. It also reveals to lower the fatty acid and triglyceride concentrations [16]. The probable molecular mechanisms of metformin function are not fully clarified. Metformin enhances AMPK expression in the liver [17], normally by an upstream kinase mediator known as LBK1 [18]. However, Foretz et al. confirmed a preserved glucose-repressing effect even though it is non-existent in either AMPK or LKB1 in hepatocytes through animal studies [19]. Metformin does not directly modulate AMPK function, but there is evidence that enhancing AMPK expression is secondary to the effect of metformin in the mitochondria [20, 21]. The function of metformin does not necessitate the AMPK activation; instead, AMPK is enhanced by metabolic stresses that increase the intracellular ADP/ATP and AMP/ADP ratio, and this might be a consecutive explanation of how metformin activates AMPK [22-24]. Despite the well-reported anti-diabetic effects of metformin, few studies have demonstrated the protective mechanisms of metformin against oxLDL-induced endothelial dysfunction as well as the anti-atherosclerotic ability.

Previous studies have suggested that the metformin increases SIRT1 and represses the pro-inflammatory state in patients with carotid artery atherosclerosis, which suggests that metformin might be an SIRT1 activator [25]. In the present study, we investigated the underlying mechanism of the effect of metformin on the oxLDL-induced endothelial dysfunction. Additionally, we hypothesized that metformin reduces the oxLDL-induced endothelial death through SIRT1 activation, thereby enhancing both AMPKα expression and NO bioavailability in human endothelial cells.

## RESULTS

### Metformin increases SIRT1 expression through AMPKα independent

**Figure 1:**
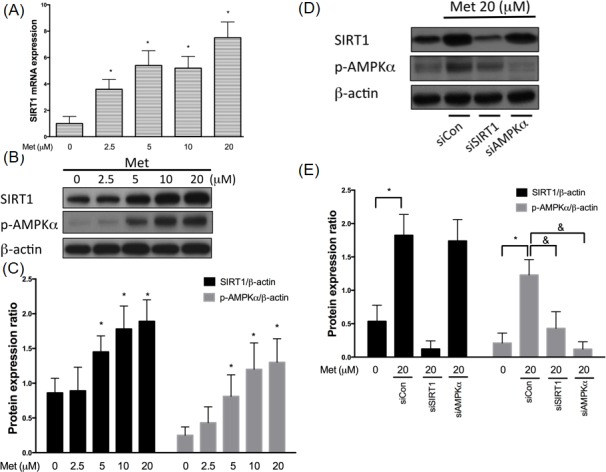
Metformin enhances SIRT1 expression through AMPK independent mechanism HUVECs were pretreated with metformin (2.5-20 μM) for 24 hrs. SIRT1 mRNA were analyzed by real-time PCR assay **A.**, respectively. SIRT1 mRNA expression was normalized to the level of β-actin. SIRT1 and phosphorylated AMPK were tested by Western blotting assay. The protein levels of SIRT1 were normalized to the level of β-actin **B.**,**C.**. HUVECs were transfected with SIRT1 or AMPKα siRNAs for 48 hrs and followed by exposure to 20 μM metformin for 24 hrs **C.**,**D.**. At the end of the incubation period, levels of both SIRT1 and phosphorylated AMPKα were determined by immunoblotting. Data are mean±SD of three different experiments. *p<0.05 compared with untreated control HUVECs. &p<0.05 compared with siControl.

SIRT1 is a known protector that delays cardiovascular diseases [26]. In Figure 1, we confirmed that metformin incubation activated SIRT1 mRNA expression levels using real-time PCR (Figure 1A). We also have shown that both SIRT1 and phosphor-AMPKα expression levels were enriched after 2.5-5 μM metformin exposure for 24 hrs in the human endothelial cells (Figure 1B, 1C). Since metformin is an AMPKα activator, we used siRNAs to clarify if increases SIRT1 expression by modulating AMPKα. In Figure 1D and 1E, we found that metformin-enhanced AMPKα phosphorylation was partially diminished by SIRT1 siRNA. In addition, silencing SIRT1 caused a significant inhibition of metformin-enhanced AMPKα phosphorylation in the human endothelial cells. Taken together, SIRT1 might act as an upstream mediator to regulate the AMPKα function in the metformin-caused AMPKα activation.

### Metformin represses oxLDL-mitigated AMPKα expression involving of SIRT1

**Figure 2:**
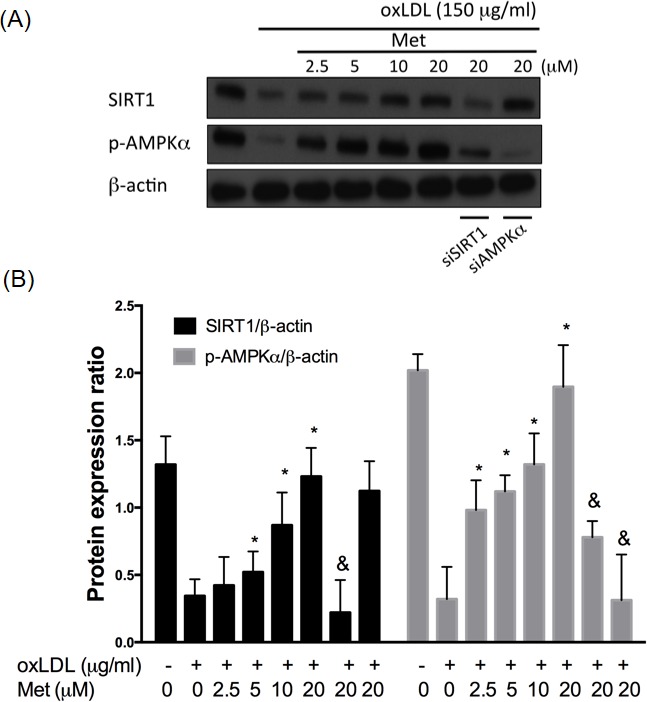
Metformin protects against oxLDL-impaired SIRT1 level HUVECs were pretreated with metformin (2.5-20 μM) for 2 hrs, followed by exposure to oxLDL (150 μg/mL) for another 24 hrs. At the end of the incubation period, cells were lysed and SIRT1 protein were analyzed by Western blotting, respectively **A.**, **B.**. In some cases, HUVECs were transfected with SIRT1 or AMPKα siRNAs for 48 hrs then treated with 20 μM of metformin followed by exposure to 150 μg/mL oxLDL for 24 hrs. The protein levels of SIRT1 and phosphorylated AMPKα were normalized to the level of β-actin. Data are mean±SD of three different experiments. #p<0.05 compared with untreated control HUVECs. *p<0.05 compared with oxLDL-stimulated HUVECs. &p<0.05 compared with oxLDL+metformin treatment.

In order to confirm the protective effect of metformin on oxLDL-impaired SIRT1 expression, total protein samples from endothelial cells exposed to oxLDL were investigated. In Figure 2A and 2B, endothelial cells exposing to oxLDL reduced SIRT1 expression and phosphor-AMPKα levels significantly compared with those of the control cells. As expected, the pre-treatment of metformin protected against oxLDL-impaired phosphorylation of AMPKα and SIRT1 expression in a dose-dependent manner. In addition, silencing AMPKα did not collapse the protective effect of metformin on oxLDL-impaired SIRT1 expression. On the other hand, the inhibitory effect of metformin on oxLDL-reduced AMPKα phosphorylation was reversed by siSIRT1, which suggested that metformin repressed the oxLDL-mitigated AMPKα expression through the modulation of SIRT1.

### Metformin reduces oxLDL-activated PKCα and NADPH oxidase activity

**Figure 3:**
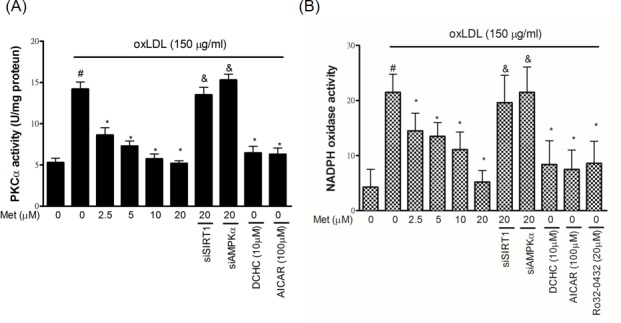
Metformin protects against oxLDL-induced PKCα and NADPH oxidase activation in endothelial cells HUVECs were pretreated with metformin (2.5-20 μM) for 2 h, followed by exposure to oxLDL (150 μg/mL) for a further 2 h. In some cases, various pharmacological activators such as SIRT1 activator (DCHC) and AMPK activator (AICAR) were added before oxLDL stimulation. Some HUVECs were transfected with SIRT1 or AMPKα siRNAs for 48 hrs then treated with 20 μM of metformin followed by exposure to 150 μg/mL oxLDL for 24 hrs. **A.** PKCα activity was tested by PKCα activity assay. PKCα inhibitor (Ro-32-0432) was used to confirm whether oxLDL-caused NADPH oxidase activation by PKCα up-regulation. **B.** NADPH oxidase activity was tested by NADPH oxidase activity assay. Data are mean±SD of three different experiments. #p<0.05 compared with untreated control HUVECs. *p<0.05 compared with oxLDL-stimulated HUVECs. &p<0.05 compared with oxLDL+metformin treatment.

Both SIRT1 and AMPKα are able to modulate and control different cellular signaling transduction pathways to reduce endothelial oxidative damage including one of the major targets, protein kinase Cα (PKCα). We previously showed that oxLDL stimulation increased the PKCα activity, thereby facilitating endothelial cell death [27]. As revealed in Figure 3A, metformin reduced the oxLDL-caused up-regulation of PKCα activity in HUVECs in a dose-dependent result. However, the protective ability of metformin on oxLDL-increased PKCα activity was significantly crashed by siSIRT1 and siAMPKα. The pre-treatments of SIRT1 activator (DCHC) and AMPKα activator (AICAR) also reduced oxLDL-increased PKCα activity. Moreover, PKC isoforms play a key role in controlling the NADPH subunit expression. In particular, the translocation of p47phox from the cytosol fraction to the membrane fraction [28], and AMPKα can mitigate free radical formation through inhibition of protein kinase C (PKC), which in turn attenuates the activation of NADPH oxidase [29]. We, therefore, sought to investigate whether metformin modulates oxLDL-induced NADPH oxidase activation. As shown in Figure 3B, the pre-treatment of oxLDL-exposed cells with metformin cause a dose-dependent inhibition in NADPH oxidase activity. Moreover, we also confirmed that silencing both SIRT1 and AMPKα with siRNAs collapsed this finding. As expected, the activation of SIRT1 and AMPKα by activators conspicuously inhibited oxLDL-caused NADPH oxidase activation. Additionally, the PKCα inhibitor, Ro32-0432, reduced oxLDL-increase NADPH oxidase activity.

### Metformin reduces oxLDL-caused oxidative stress

**Figure 4:**
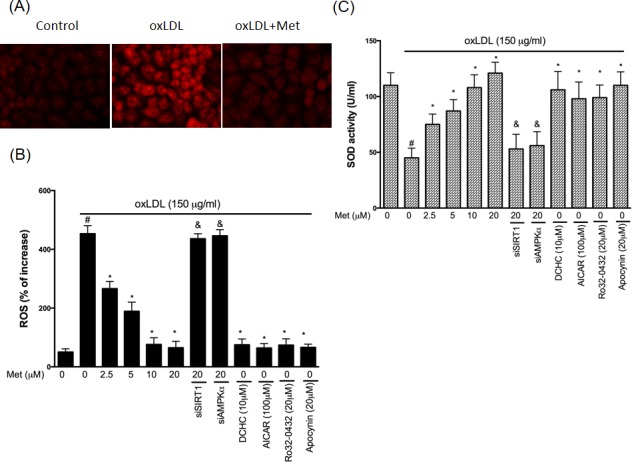
Metformin repressed oxLDL-induced oxidative stress in endothelial cells Cells were treated with 150 μg/mL oxLDL for 24hrs followed by a incubation with superoxide-sensitive fluorescent probe DHE (10 μM) with or without metformin treatment. In some cases, various pharmacological drugs such as SIRT1 activator (DCHC) and AMPK activator (AICAR), PKCα inhibitor (Ro-32-0432) and NADPH oxidase inhibitor (Apocynin) were added before oxLDL stimulation. Some HUVECs were transfected with SIRT1 or AMPKα siRNAs for 48 hrs then treated with 20 μM of metformin followed by exposure to 150 μg/mL oxLDL for 24 hrs. **A.** Fluorescence images show the ROS level in control cells and HUVECs stimulated with oxLDL alone and in the presence of metformin. **B.** Fluorescence intensity of cells was measured with a fluorescence microplate reader. Fluorescence distribution of DHE oxidation is expressed as a percentage of increased intensity. **C.** The activity of SOD and in HUVECs stimulated with oxLDL in were determined. Data are mean±SD of three different experiments. ♯p<0.05 compared with untreated control HUVECs. *p<0.05 compared with oxLDL-stimulated HUVECs. &p<0.05 compared with oxLDL+metformin treatment.

Up-regulation of PKCα and NADPH oxidase were thought to increase endothelial oxidative stress by increasing the intracellular ROS generation. In Figure 4A and 4B, endothelial ROS levels were increased by oxLDL stimulation for 24hrs. However, the pre-treatment of metformin reduced oxLDL-generated intracellular ROS. The knockdown of SIRT1 and AMPKα using siRNAs impaired the protective effects of metformin. The pharmacological SIRT1 activator (DCHC), AMPKα activator (AICAR), PKCα inhibitor and NADPH oxidase inhibitor also reduced the oxLDL-increased ROS concentrations. Next, we investigated the superoxide dismutase (SOD) enzymic activity to understand the mechanisms of metformin's antioxidant ability in the HUVECs stimulated to oxLDL. In Figure 4C, we revealed that the HUVECs with metformin pre-treated superoxide dismutase was largely potentiated. In consistency with our previous data, the pre-treatments with pharmacological SIRT1 activator (DCHC), AMPKα activator (AICAR), PKCα inhibitor and NADPH oxidase inhibitor reversed the oxLDL-repressed antioxidant enzymes function. However, metformin protecting against oxLDL-reduced SOD activity was collapsed by both silencing SIRT1 and AMPKα.

### Metformin reduces oxLDL-caused LOX-1 overexpression

**Figure 5:**
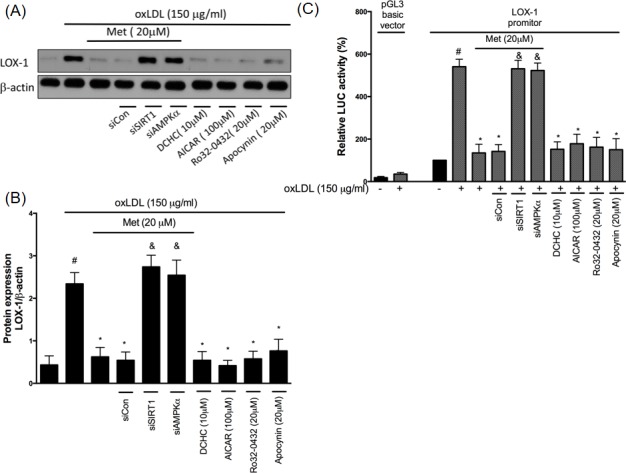
Metformin protects against oxLDL-induced LOX-1 up-regulation HUVECs were pretreated with metformin for 2 hrs, followed by exposure to oxLDL (150 μg/mL) for another 24 hrs. At the end of the incubation period, cells were lysed and SIRT1 protein were analyzed by Western blotting, respectively **A.**, **B.**. In some cases, HUVECs were transfected with SIRT1 or AMPKα siRNAs for 48 hrs then treated with 20 μM of metformin followed by exposure to 150 μg/mL oxLDL for 24 hrs. In some causes, pharmacological drugs such as SIRT1 activator (DCHC), AMPK activator (AICAR), PKCα inhibitor (Ro-32-0432) and NADPH oxidase inhibitor (Apocynin) were added before oxLDL stimulation. The protein levels of LOX-1 were normalized to the level of β-actin. **C.** HUVECs were transfected with the LOX-1 promoter. After 24 h of transfection, HUVECs were stimulated with oxLDL with pr without pharmacological drugs for an additional 24 hrs. The total cell lysates were tested using a luciferase (LUC) activity assay. Data are mean±SD of three different experiments. #p<0.05 compared with untreated control HUVECs. *p<0.05 compared with oxLDL-stimulated HUVECs. &p<0.05 compared with oxLDL+metformin treatment.

The lectin-like oxLDL receptor (LOX-1) has been recognized as an important receptor for oxLDL. In the human endothelial cells, it is well known that the binding of oxLDL to LOX-1 rapidly increases ROS concentrations by the up-regulation of NADPH oxidase activity. In addition, the activated oxidase stress could then further enhance the LOX-1 level [30]. Previously, we found that oxidative stress causes LOX-1 up-regulation through NADPH oxidase activation [31]. In Figure 5, we found that the pre-treatment of metformin can reduce oxLDL-induced LOX-1 up-regulation. However, this phenomenon was reversed by both siSIRT1 and siAMPKα. DCHC, AICAR, Ro32-0432 and Apocynin also significantly reduced oxLDL-caused LOX-1 over-expression. Thus, we suggested that metformin protects against oxLDL-induced LOX-1 up-regulation involving the SIRT1/AMPKα/PKCα/NADPH oxidase pathway. This hypothesis was also supported by employing the LOX-1 promoter assay (Figure 5C).

### Metformin-mediated protection involves AKT/eNOS activation

**Figure 6:**
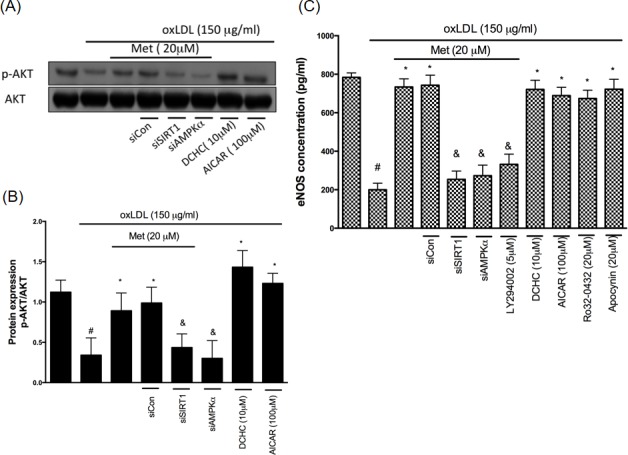
Metformin protects against oxLDL-repressed AKT and eNOS expression HUVECs were pretreated with metformin for 2 hrs, followed by exposure to oxLDL (150 μg/mL) for another 24 hrs. At the end of the incubation period, cells were lysed and SIRT1 protein were analyzed by Western blotting, respectively **A.**, **B.**. In some cases, HUVECs were transfected with SIRT1 or AMPKα siRNAs for 48 hrs then treated with 20 μM of metformin followed by exposure to 150 μg/mL oxLDL for 24 hrs. In some causes, pharmacological drugs such as SIRT1 activator (DCHC) and AMPK activator (AICAR) were added before oxLDL stimulation. The protein levels of phosphorylated AKT were normalized to the level of AKT. **C.** eNOS expression levels were tested by ELISA assay. Data are mean±SD of three different experiments. #p<0.05 compared with untreated control HUVECs. *p<0.05 compared with oxLDL-stimulated HUVECs. &p<0.05 compared with oxLDL+metformin treatment.

AKT is known as the main protector in maintaining the cell survival in response to different attractions as well as enhancing the activity of endothelial nitric oxide synthase (eNOS) that trigger the nitric oxide (NO) generation. Previous reports have shown that the treatment of oxLDL impaired AKT phosphorylation and eNOS activity in endothelial cells by the LOX-1 activation [27]. On the other hand, the activation of AKT and eNOS was revealed to protect against cell death [32]. In order to understand whether LOX-1/AKT/eNOS mechanism is included in the protective effects of metformin, we performed a Western blot analysis with utilizing the phosphor-specific AKT antibody (Figure 6A and 6B) and eNOS expression (Figure 6C). Results from Figure 6 indicated that metformin significantly reversed the oxLDL-induced de-phosphorylation of AKT and deactivate of eNOS. This phenomenon was reversed by both siSIRT1 and siAMPKα. DCHC as well as AICAR significantly protected against oxLDL-caused de-phosphorylation of AKT and repression of eNOS. The AKT inhibitor (LY294002) treatment impaired the protective ability of metformin from oxLDL-caused inhibition of eNOS. In addition, Ro32-0432 and Apocynin repressed oxLDL-impaired eNOS expression.

### Metformin protects against oxLDL-induced intracellular calcium rise and mitochondrial dysfunction

**Figure 7:**
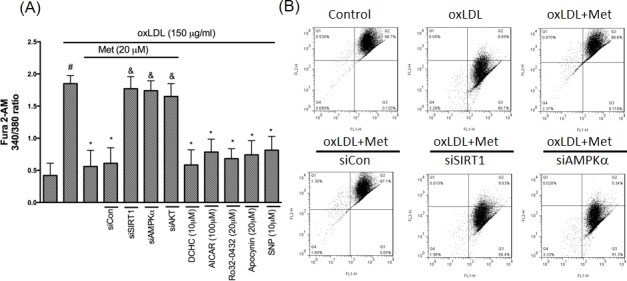
Metformin represses oxLDL-elevated Ca^2+^ and prevents mitochondrial dysfunction **A.**. HUVECs were pretreated with imetformin for 2 hrs followed by stimulation with oxLDL (150 μg/ml) for another 24 hrs. In some cases, HUVECs were transfected with SIRT1 or AMPKα siRNAs for 48 hrs then treated with 20 μM of metformin followed by exposure to 150 μg/mL oxLDL for 24 hrs. In some causes, pharmacological drugs such as SIRT1 activator (DCHC), AMPK activator (AICAR), NO donor (SNP), PKCα inhibitor (Ro-32-0432) and NADPH oxidase inhibitor (Apocynin) were added before oxLDL stimulation. The measurement of intracellular calcium is described in the Materials and Methods. **B.** ΔΨm was inspected with the signal from monomeric and J-aggregate JC-1 fluorescence, as described in the Materials and Methods. JC-1 fluorescence was confirmed by flow cytometry. Data are mean±SD of three different experiments. #p<0.05 compared with untreated control HUVECs. *p<0.05 compared with oxLDL-stimulated HUVECs. &p<0.05 compared with oxLDL+metformin treatment.

LOX-1 also acts an important role in modulating NADPH oxidase/AKT/eNOS and Ca^2+^ signaling pathway [33]. Therefore, we investigated the protective effect of stimulating endothelial cells to a harmful concentration of oxLDL on intracellular calcium concentration and exposed endothelial with oxLDL in the absence or presence of metformin. Clearly, we found that the 340/380 ratio of [Ca^2+^]_i_ up-regulated in oxLDL-attracted HUVECs was repressed in the HUVECs pretreatment of metformin. Silencing of SIRT1, AMPKα and AKT failed the cyto-protective function of metformin. Moreover, DCHC, AICAR, Ro32-0432, Apocynin and NO donor (SNP) significantly inhibited the oxLDL-elevated Ca^2+^ concentration (Figure 7A). The elevation in intracellular Ca^2+^ caused the up-regulation of different calcium-dependent pro-apoptotic events. Specifically, intracellular Ca^2+^ is the key regulator for initiating the mitochondrial permeability transition pore (PTP) for the reason that it causes cell death. As a consequence of both the dysfunction of the electrochemical gradient induced by pore opening and rupture of the outer mitochondrial membrane, the mitochondrial membrane potential (Ψm) generally collapses. Then, we investigated the mitochondrial permeability to determine whether metformin preserves the mitochondrial stability after attracting to oxLDL. In Figure 7B, oxLDL depolarized the mitochondrial transmembrane potential in endothelial cells as shown by the increase of the green fluorescence. However, metformin led to the maintenance of mitochondrial transmembrane potential in which the repression of green fluorescence and restoration of red fluorescence were indicated. Silencing of SIRT1, AMPKα failed the cyto-protective function of metformin.

### Metformin protects against oxLDL-induced endothelial apoptosis

**Figure 8:**
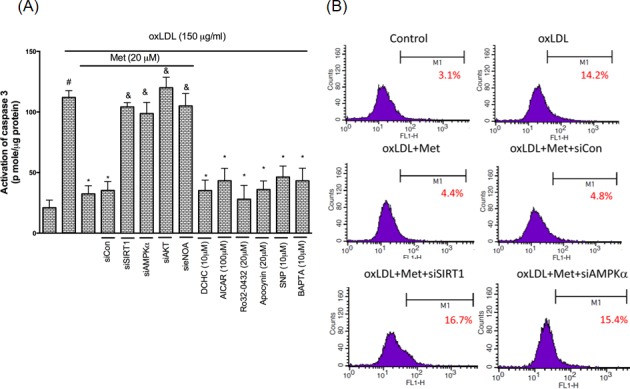
Metformin prevents oxLDL-induced endothelial apoptosis HUVECs were incubated for 1 hr with Metformin, followed by exposure to oxLDL (150 μg/ml) for another 24 hrs. In some cases, HUVECs were transfected with SIRT1 or AMPKα siRNAs for 48 hrs then treated with 20 μM of metformin followed by exposure to 150 μg/mL oxLDL for 24 hrs. In some causes, pharmacological drugs such as SIRT1 activator (DCHC), AMPK activator (AICAR), NO donor (SNP), PKCα inhibitor (Ro-32-0432), NADPH oxidase inhibitor (Apocynin) and Ca^2+^ inhibitor (BAPTA) were added before oxLDL stimulation. The activity of caspase 3 was measured by EnzCaspase-3 assay kit **A.** Apoptotic cells were detected using a TUNEL assay **B.** Data are mean±SD of three different experiments. #p<0.05 compared with untreated control HUVECs. *p<0.05 compared with oxLDL-stimulated HUVECs. &p<0.05 compared with oxLDL+metformin treatment.

In regards to explain if metformin modulates the activity of the apoptotic regulators in controlling pro-apoptotic events, we investigated the influence of metformin on oxLDL-promoted activation of caspase 3 using the EnzCaspase-3 activity kit. It was demonstrated that metformin protected against oxLDL-caused caspase 3 activation. The concomitant intervention of metformin and the SIRT1, AMPKα, NO donors reduced oxLDL-caused caspase 3 activation. On the other hand, the pre-treatment of PKCα, NADPH oxidase and Ca^2+^ inhibitors also definitely reduced oxLDL-caused caspase 3 activation. However, silencing of SIRT1, AMPKα and AKT abolished the cyto-protective ability of metformin (Figure 8A). Lastly, the anti-apoptotic function of metformin was further confirmed by using the TUNEL assay (Figure 8B). Basically, our results indicated that the modulation of SIRT1/AMPKα pathway through PKCα/NADPH oxidase/LOX-1/AKT/NO/Ca^2+^ mechanism metformin repressed the oxLDL-caused endothelial apoptosis.

## DISCUSSION

The increased oxidative stress and the formation of ROS like superoxide act as key modulators in the development of pro-atherosclerotic and anti-atherosclerotic processes in the human endothelial wall. This study has elucidated the probable mechanism whereby metformin protects against the oxLDL-induced human endothelial dysfunction. In this present study, we successfully revealed that the metformin pre-treatment attenuates oxLDL-caused endothelial apoptosis. Metformin enhanced SIRT1 expression in both protein and mRNA levels and promoted phosphorylation of AMPK, thereby contributing the potent protective abilities against oxidative injuries. The signaling pathways of metformin-mediated anti-atherosclerotic effects involve inhibit the NADPH oxidase activity as well as the ROS formation, which therefore mitigates the LOX-1 up-regulation and AKT/eNOS deactivation. In addition, metformin suppressed both Ca^2+^ elevation and mitochondrial dysfunction by the oxLDL induction in the human endothelial cells.

Furthermore, SIRT1 is recognized as a novel target for prohibiting the early stage atherosclerosis in the human endothelial cells. The SIRT1 gene is a member of nicotinamide adenine dinucleotide (NAD)-dependent genes and is recognized as a key protector against oxidative injuries, apoptosis, pro-inflammatory events and cardiovascular degeneration [34]. Enhancing the SIRT1 function mitigates ischemia/reperfusion-induced cardiac hypertrophy and myocardial dysfunction [35]. In addition, SIRT1 modulates the human endothelial vessel function and reduces macrophage foam cell generation in the human vessel cells [36]. A previous study indicated that silencing the SIRT1 expression causes premature senescence-like growth arrest in the HUVECs, whereas the up-regulation of SIRT1 function presents premature senescence by hydrogen peroxide induction [37]. SIRT1 also modulates the human endothelial function by reducing foam cell generation and calcification [36]. Moreover, it also represses the pro-inflammatory responses by directly inhibiting the nuclear factor (NF)-κB activity [38]. In addition, SIRT1 de-acetylates LKB1 that results to increase the AMPK expression, which is known as a main controller involved in energy homeostasis; this indicates the SIRT1-LKB1-AMPK mechanism contributing to energy imbalance, cellular dysfunction and initiation of pro-apoptotic events [34]. In the present study, we revealed that the metformin treatment promotes SIRT1 expression levels by AMPKα independent pathway. However, metformin enhances AMPKα phosphorylation that involves with the SIRT1 (Figure 1). We also suggested that the modulation of SIRT1 promotes metformin to reverse oxLDL-impaired SIRT1 and AMPKα phosphorylation (Figure 2).

Particularly, PKCα is one of the main modulators of the cellular physiological function under oxidative stimulation. We previously indicated that PKC activation acts as the main role in oxLDL-caused endothelial inflammation and cell death [27]. PKC knockout mice delayed the progression of atherosclerotic damages [39]. Nowadays, this finding indicates that PKC modulates both endothelial apoptosis and proliferation during the development of atherosclerosis. On the other hand, PKC-specific pharmacological inhibitor mitigates the stress-caused oxidative injuries and mitochondrial dysfunction in endothelial cells that had been reported, which suggests that PKC inhibition might be a good strategy to prevent atherosclerosis [40]. AMPK has been identified to inhibit human abnormality of a vessel under oxidative injuries through modulating the AKT/eNOS mechanism as well as mitigating the PKC-drived activation of NADPH oxidase in the human endothelial tissues [29, 41]. In this study, we reported that the regulation of SIRT1/AMPKα provoked metformin to reduce the oxLDL-enriched PKCα activity (Figure 4A). Also, it was demonstrated that metformin reduced theoxLDL-caused NADPH oxidase activation, but, silencing SIRT1 and AMPKα impaired this outcome (Figure 4B).

In general, oxidative attracting is a major reason that contributes to atherosclerotic events. The up-regulated ROS concentration causes human endothelial dysfunction. Under oxidative stimulation, NADPH oxidase plays an important role to turn on oxidative signaling transduction by the generation of ROS *via* assembly with NADPH oxidase subunits [28]. Moreover, few new studies have reported considerable benefits of antioxidants for the management and therapeutic approach on atherosclerosis [42]. SIRT1 activation enriches the function of antioxidant enzymes such as SOD, catalase, as well as glutathione peroxidase (GSH), thereby mitigating the oxidative damages [43]. Therefore, we successfully confirmed the metformin treatment reduces oxLDL-facilitated ROS generation as well as oxLDL-impaired SOD activity (Figure 4) by modulating the SIRT1/AMPKα axis.

Previously, the LOX-1 was reported as a receptor for oxLDL [44]. The up-regulation of LOX-1 expression promptly facilitates ROS generation, NADPH oxidase activation [45] and atherosclerotic lesions [46]. By using the Western blotting assay and LOX-1 promoter assay, we identified that the metformin treatment repressed oxLDL-caused LOX-1 up-regulation through the SIRT1/AMPKα/PKCα/NADPH oxidase mechanism (Figure 5). Previous reports have shown that the activated LOX-1 level would be able to repress the AKT expression by up-regulating the p38 mitogen-activated protein kinase (MAPK) and inhibiting the NO secretion through impairing endothelial NO synthase (eNOS) [47]. Stein et al. reported that SIRT1 delays cardiovascular degeneration by activating eNOS [48]. In this present study, we had confirmed that oxLDL-impaired AKT phosphorylation and eNOS activity. However, the metformin treatment reversed oxLDL-impaired AKT and eNOS function through SIRT1/AMPKα and PKCα/NADPH oxidase mechanism (Figure 6).

Furthermore, the maintenance of SIRT1 homeostasis is fundamental to inhibit the harmful mechanisms of atherosclerosis such as the forkhead transcription factors (FoxOs). For example, SIRT1 de-acetylated both FoxO 1 and FoxO3 in which reduces DNA damage, cell cycle arrest and oxidative injuries [49]. Both AKT and eNOS cooperate to maintain the mitochondria normal function and to prevent apoptosis under oxidative stimulation as reported in a previous study [50].

Moreover, metformin is one of the most extensively used anti-diabetic medications by enhancing the insulin sensitivity in patients with type 2 diabetes (Stumvoll et al., 1995). In addition, metformin mitigates the hazard of athero-thrombotic dysfunction accompanied with diabetes disconnected of its antihyperglycemic ability [51]. Suparna et al. suggested that metformin ameliorates the endothelial performance locating in the aortic tissue on diabetic hyperglycemic stimulations [52]. It reduces diabetic lipo-apoptosis in the human coronary artery endothelial cells through the activation of AMPK/eNOS and MAPK kinase. Specifically, metformin is an activator of AMPK [53]. Recently, it was considered as an SIRT1 agonist. The metformin treatment alleviates hyperglycemia-caused senescence and cell death from modulating SIRT1 in the endothelial cells [54]. The physiological concentrations of metformin were investigated. Subjects were intaking oral administration of 500 mg metformin three times daily for 21 days. The physiological concentrations of metformin were 5.9 ± 0.8 to 8.7 ± 0.7 μmol/L. In this present study, the pre-treatment with metformin 5 μM, a physiological attainable concentration, was sufficient to protect oxLDL-induced SIRT1and AMPK dysfunction, which suppressed the oxLDL-Facilitated NADPH oxidase activation and attenuated the activation of the LOX-1-mediated oxidative signaling pathway. Therefore, the metformin treatment for inhibiting oxLDL-induced oxidative injuries is clinically available.

In conclusion, we confirmed here for the first time that metformin inhibits the oxLDL-caused SIRT1 mitigation that is closely correlated to the human vessel dysfunction. It mainly reversed the oxLDL-caused AMPK-α de-activation and oxLDL-activated PKCα and NADPH oxidase expression, thereby repressing the ROS formation and antioxidant enzyme dysfunction. Metformin enhances the AKT/eNOS expression under oxLD stimulation, which also represses the LOX-1-mediated oxidative signaling in the endothelial cells. This present report was performed *in vitro*, and further investigations are required to confirm the extent to which metformin can repress the oxLDL-facilitated pro-atherogenic functions and metformin efficacies *in vivo*. In closing, our comprehensive findings provided additional evidences that metformin provides beneficial effects on cardiovascular health.

## MATERIALS AND METHODS

### Cell culture and reagents

Human umbilical vein endothelial cells (HUVECs) were obtained from ATCC. HUVECs were cultured with M199 basal medium supplemented with low-serum growth supplement and penicillin (50 IU/ml)-streptomycin (50 μg/ml). Trypsin-EDTA was used to passage cells. M199 and trypsin-EDTA were obtained from Gibco (Grand Island, NY, USA). Low-serum growth supplement was purchased from Cascade (Portland, OR, USA). Additionally, 5,58,6,68-tetraethylbenzimidazolcarbocyanine iodide (JC-1) were purchased from BioVision (Palo Alto, CA, USA). LY294002, dihydroethidium (DHE), Apocynin, 1,2-bis(o-aminophenoxy)ethane-N,N,N′,N′-tetraacetic acid (BAPTA), DCHC, AICAR, sodium nitroprusside (SNP), Ro-320432, penicillin and streptomycin were all purchased from Sigma (St. Louis, MO, USA). Anti-β-actin, anti-AMPK, anti-AMPK-α, anti-AKT, anti-phospho AKT, anti-LOX-1, anti-SIRT1, anti-eNOS were all obtained from Santa Cruz Biotechnology (Santa Cruz, CA, USA). HRP-conjugated anti-rabbit secondary antibodies were purchased from Transduction Laboratories (CA, USA). (TUNEL) staining kit was obtained from Boehringer Mannheim (Mannheim, Germany) was purchased from Selleckchem. Fura-2 AM and the EnzChek caspase 3 assay kit were purchased from Molecular Probes (Eugene, OR). SOD activity assay kit was obtained from Calbiochem (San Diego, CA). eNOS kit was purchased from R&D Systems (Minneapolis, MN, USA).

### Lipoprotein separation

The protocol for LDL separation used in this study has been previously described [55] Briefly, native LDL was isolated from fresh normolipidemic human serum by sequential ultracentrifugation (*ρ* = 1.019-1.210 g/ml) in KBr solution containing 30 mM EDTA. Immediately before oxidation, LDL was separated from EDTA and also from diffusible low molecular mass compounds by gel filtration on PD-10 Sephadex G-25 M gel (Pharmacia, St-Quentin, France) in 0.01 M phosphate-buffered saline (PBS; 136.9 mM NaCl, 2.68 mM KCl, 4 mM Na2HPO4, 1.76 mM KH2PO4, pH 7.4). Copper-modified LDL (1 mg protein/ml) was prepared by exposing LDL to 10 μM CuSO4 for 16 hrs at 37°C. The oxLDL that has been studied had a TBARS value of 16-20 nM/mg protein of LDL protein; native LDL had no detectable TBARS.

### Measurement of ROS production

The effect of metformin on ROS production in HUVECs was determined by DHE. Confluent HUVECs (104 cells/well) in a 96-well plates were pre-incubated with various concentrations of metformin for 2 hrs. oxLDL was then added to the medium in the absence or presence of metformin for an additional 2 hrs. After removing the medium from the wells, the cells were incubated with 10 μM DHE for about one hr. The fluorescence intensity was measured with a fluorescent microplate reader (Labsystem, CA, USA) calibrated with an excitation at 540 nm and emission at 590 nm. The increased percentage in fluorescence per well was calculated by the formula [(Ft2−Ft0)/Ft0]×100, which Ft2 is the fluorescence at 2 hrs of oxLDL exposure, and Ft0 is the fluorescence at 0 hr of oxLDL exposure.

### Transfection with small interfering RNA (siRNA)

On-target Plus SMART pool siRNAs for non-targeting control and SIRT1, AMPK-α, AKT, eNOS were purchased from Dharmacon. Two days after transfection, cells were treated with a reagent as indicated for further experiments.

### Isolation of mRNA and quantitative real-time PCR

Total RNA was isolated from HUVECs using the RNeasy kit (Qiagen, Valencia, CA, USA). Oligonucleotides for SIRT1 and β-actin were designed using the computer software package Primer Express 2.0 (Applied Biosystems, Foster City, CA, USA). All of the oligonucleotides were synthesized by Invitrogen (Breda, The Netherlands). Oligonucleotide specificity was determined by a homologous search within the human genome (BLAST, National Center for Biotechnology Information, Bethesda, MD, USA) and confirmed by the dissociation curve analysis. The oligonucleotide sequences were as followed: SIRT1 sense primer, 5′-TGTGGTAGAGCTTGCATTGATCTT-3′; anti-sense primer, 5′-GGCCTGTTGCTCTCCTCAT-3′; β-actin sense primer, 5′-AGGTCATCACTATTGGCAACGA-3′; anti-sense primer, 5′-CACTTCATGATGGAATTGAATGTAGTT-3′. PCR was performed with SYBR Green in an ABI 7000 sequence detection system (Applied Biosystems) according to the manufacturer's guidelines.

### NADPH oxidase activity assay

The lucigenin method was used to determine the NADPH oxidase activity in HUVECs. The crude membrane fraction of HUVECs were obtained as mentioned earlier. The total protein concentration was adjusted to 1 mg/ml. An aliquot 200 μl of protein (100 μg) was incubated in the presence of 5 μM lucigenin and 100 μM NADPH. The luminescence was measured after 10 minutes by a plate reader (VICTOR3; Perkin-Elmer) to determine the relative changes in NADPH oxidase activity.

### Promoter activity assay

The Dual Luciferase Reporter Assay System (Promega) was used to test the luciferase activity. The luciferase intensity was measured using a luminometer. The relative intensities were measured by normalizing with a Renilla luciferase activity for the efficiency of transfection.

### Immunoblotting

After stimulating the oxLDL of HUVECs, cells were washed, scraped from dishes, and lysed in the RIPA buffer. Proteins were then separated by electrophoresis on a SDS-polyacrylamide gel. After the proteins had been transferred onto a PVDF membrane, the blot was incubated with a blocking buffer for one hr at room temperature and then probed with primary antibodies overnight at 4°C, then followed by incubating with horseradish peroxidase-conjugated secondary antibody for another one hr.

### SOD activity

The SOD levels were tested *via* an enzymatic assay method using commercial kits (Sigma) according to the manufacturer's instructions.

### eNOS assay

At the end of the oxLDL incubation period, cells were washed, scraped from dishes, and lysed in the RIPA buffer. Total protein was for eNOS expression using an ELISA kit obtained from R&D Systems. The data are expressed in nanograms per milliliter of duplicate samples.

### Measurement of [Ca2+]i

To determine the effect of metformin on the oxLDL-induced increase in intracellular calcium concentration, HUVECs were seeded onto 24-mm glass coverslips, pretreated with CoQ10 for 2 hrs and then stimulated with oxLDL for 24 hrs. The cells on the coverslips were loaded with 2 μM fura-2 AM (Molecular Probe) in M199 and allowed to stand for 30 min at 37°C. After loading, the cells were washed with PBS to remove excess fluorescent dye. Then, the fluorescence of the cells on each coverslip was measured and recorded using an inverted Olympus microscope IX-70. [Ca2+]i in endothelial cells was monitored at an emission wavelength of 510 nm with excitation wavelengths alternating between 340 and 380 nm with the use of a cooled charge-coupled device (CCD) camera (MicroMAX, 782YHS; Roper Scientific, Trenton, NJ), recorded using SimplePCI 6.0 (Compix Institute, Cranberry Township, PA) and calculated using Grynkiewicz's method.

### Measurement of mitochondrial membrane potential

The lipophilic cationic probe fluorochrome 5,58,6,68-tetraethylbenzimidazolcarbocyanine iodide (JC-1) was used to explore the effects of metformin on mitochondria membrane potential (ΔΨm). JC-1 exists either as a green fluorescent monomer at depolarized membrane potentials or as a red fluorescent J-aggregate at hyperpolarized membrane potentials. After treating cells with oxLDL (150 μg/ml) for 24 hrs in the presence or absence of metformin cells, they were rinsed with M199 and JC-1 (5 μM) was then loaded after. After 20 minutes of incubating at 37°C, cells were examined under a fluorescent microscope.

### Investigation of apoptosis

Apoptotic cells that were assessed with the TUNEL assay were visualized under a fluorescence microscope or analyzed by flow cytometry. HUVECs were cultured to confluent on glass slides. After treatment, HUVECs were rinsed twice in PBS before fixation for 30 min at room temperature with 4% paraformaldehyde. Next, the slides were washed in PBS before incubating in the prepared solution (0.1% Triton X-100, 0.1% sodium citrate) for 5 min. The slides were then incubated with 100 μl TUNEL reaction mixture in a humidified atmosphere for one hr at 37°C in the dark, washed in PBS, and counterstained with propidium iodide.

### Measurement of active caspase 3

HUVECs were pretreated with metformin for 2 hrs and then stimulated with oxLDL (150 μg/ml) for 24 hrs. In some cases, HUVECs were incubated with metformin. The activity of caspase 3 was measured by an EnzChek caspase-3 assay kit according to the manufacturer's instructions (Molecular Probes). After being lysed by repeated freeze-thaw cycles, equal amounts of protein (50 μg) were added to the reaction buffer containing 5 mM of caspase 3 substrate Z-DEVD-R110, and the mixture was incubated at room temperature for 30 min. The fluorescence generated from the cleavage of the substrate by caspase 3 was monitored with a fluorescence microplate reader (Labsystems) calibrated for excitation at 496 nm and for emission at 520 nm.

### Statistical analyses

The results are expressed as means ± SD. Statistical analyses were performed using one-way or two-way ANOVA followed by Tukey's post-hoc test as appropriate. A p-value<0.05 was considered statistically significant.
